# The effect of physical exercise on sleep quality in university students: chain mediation of health literacy and life satisfaction

**DOI:** 10.3389/fpsyg.2025.1604916

**Published:** 2025-07-01

**Authors:** Mo-Han He, Tian-Yu Zhao, Wei-Dong Zhu, Hu Lou, Ding-You Zhang, Fan-Zheng Mu, Xin-Yu Zhang, Yu-Han Li, Wen-Hao Zhang, Qi Liu, Jia-Qiang Wang, Chen-Xi Li, Hao-Yu Li, Ning Zhou, Yao Zhang, Hao-Jie Zuo, Wei Wang, Xiao-Yu Wang, Bo-Chun Lu, Lin-Lin Zhao, Shan-Shan Han, Ya-Xing Li, Yang-Sheng Zhang, Ling-Li Xu, Yu-Yan Qian, Chuan-Yi Xu, Han Li, Shuo Feng, Qing Zhang, Jian-Gang Sun, Lan-Lan Yang, Bo Li, Fei Gu

**Affiliations:** ^1^Institute of Sports Science, Nantong University, Nantong, China; ^2^Faculty of Nursing, Shangqiu Medical College, Shangqiu, China; ^3^School of Physical Education and Sports Science, Nantong University, Shanghai, China; ^4^School of Physical Education, Shanghai Normal University, Shanghai, China; ^5^Physical Education University, Shang Qiu University, Shangqiu, China; ^6^School of Physical Education, Nanjing Xiaozhuang University, Nanjing, China; ^7^Journal Publishing Center, Nantong University, Nantong, China; ^8^Guangxi University of Chinese Medicine, Nanning, China; ^9^Ordos Institute of Technology, Ordos, China; ^10^School of Physical Education, Xinyang Normal University, Xinyang, China; ^11^Yangling Vocational and Technical College, Yangling, China; ^12^College of Physical Education, West Anhui University, Lu'an, China

**Keywords:** physical exercise, quality of sleep, health literacy, life satisfaction, the chain mediating effect

## Abstract

**Objective:**

The present study was designed to explore the relationship between physical exercise and sleep quality. Specifically, it investigates the extent to which this relationship is mediated by a sequential process involving health literacy and life satisfaction.

**Methods:**

Data on physical exercise, sleep quality, health literacy, and life satisfaction were collected from a sample of 12,646 college students (study participants) using a questionnaire. Statistical analyses were performed using SPSS and AMOS software, including descriptive statistics, correlation analysis, and regression analysis.

**Results:**

Correlation analyses revealed a weak positive correlation between physical exercise and sleep quality (*r* = 0.290), a significant positive correlation between physical exercise and health literacy (*r* = 0.203, *p* < 0.01), and a significant positive correlation between physical exercise and life satisfaction (*r* = 0.374, *p* < 0.01). Conversely, sleep quality exhibited a significant negative correlation with health literacy (*r* = −0.091, *p* < 0.01) and a significant negative correlation with life satisfaction (*r* = −0.228, *p* < 0.01). Health literacy and life satisfaction were significantly positively correlated (*r* = 0.352, *p* < 0.01). Regression analysis indicated that physical exercise did not directly and significantly predict sleep quality (*β* = −0.010). This study exhibits a complete mediation effect. However, mediation analysis revealed a significant indirect effect of physical exercise on sleep quality through health literacy (95% CI: [−0.022, −0.013]) and life satisfaction (95% CI: [−0.024, −0.015]). Furthermore, a significant chain-mediating effect was observed, wherein physical exercise influenced sleep quality sequentially through health literacy and life satisfaction (95% CI: [−0.018, −0.013]). The non-significant direct effect of physical exercise on sleep quality and the significant indirect impact suggest that the relationship between physical exercise and sleep quality is primarily mediated through health literacy and life satisfaction. These findings highlight the crucial role of health literacy and life satisfaction as mediators in this relationship.

**Conclusion:**

While physical exercise did not exert a direct and significant effect on sleep quality in this study, the significant mediating roles of health literacy and life satisfaction suggest potential avenues for intervention. Specifically, these findings imply that multifaceted approaches, encompassing strategies to promote physical activity, enhance health literacy, and improve life satisfaction, may improve sleep quality among college students.

## Introduction

Sleep quality, defined as an individual’s subjective assessment of their sleep experience, is a multidimensional construct encompassing sleep efficiency (the ratio of time spent asleep to time spent in bed), sleep latency (the time taken to fall asleep), sleep persistence (the ability to maintain sleep throughout the night), and the frequency of arousal episodes following sleep onset (Nelson et al., 2022). As a fundamental physiological process, sleep plays an irreplaceable role in maintaining human health and well-being. However, globally, over 45% of the population experiences varying degrees of sleep quality problems (Lemoine et al., 2010); In the Chinese context, data from the 2022 China Social Mentality Survey indicate that the national average sleep duration per night is (7.40 ± 1.0) hours, with 16.79% of individuals sleeping less than 7 h. Furthermore, 10.40% of respondents self-assessed their sleep quality as “bad” or “very poor” (China Sleep Study Report 2023 Released, 2023; Yuan, 2024). This issue is particularly pronounced among college students, with studies reporting sleep disorder detection rates as high as 40.2% in this population (Fang et al., 2020). From a medical perspective, sleep quality is significantly correlated with individual health status (Vgontzas and Chrousos, 2002); Adequate sleep not only reduces disease incidence and maintains bodily homeostasis but also positively influences cognitive function, immune regulation, and metabolism (Buysse, 2014), Conversely, sleep disorders can lead to attention deficits and immune deficiency, affecting cognitive performance (Killgore, 2010); and inducing negative emotions such as anxiety. This is particularly concerning as negative emotions and reduced sleep quality often create a reinforcing, detrimental cycle (Hadi et al., 2008), compounding the negative effects on an individual’s physical and mental health.

The international community widely acknowledges the value of physical exercise as a comprehensive health promotion strategy. The World Health Organization (WHO) emphasizes that regular physical activity not only improves physical health but also effectively alleviates anxiety, depression, and other psychological challenges, thereby enhancing overall quality of life (WHO, 2021); Physical activity functions not only as a dynamic physiological process but also as a positive means of psychological adjustment, enhancing stress resistance, improving interpersonal relationships, and promoting psychological well-being (Zhang and Min, 2022), Empirical evidence supports the notion that the physiological and psychological mechanisms of physical activity facilitate the release of negative emotions, alleviate stress, and improve psychological health (Herbert et al., 2020). Despite the recognized benefits, global participation in physical activity among adults remains a concern. Data from 2016 indicate that approximately 27.5% of adults (aged 18 years and above) do not meet the WHO-recommended standard of at least 150 min of moderate-intensity or 75 min of vigorous-intensity exercise per week (Guthold et al., 2018); In China, only 30.4% of adult residents aged 19–59 years old regularly engage in physical activity (Yin et al., 2024); Of particular concern is the college student population, where the China Youth Sports Development Report (2015) indicates that the average daily physical activity time is less than 1 h, significantly below internationally recommended levels (Wang and Tang, 2024).

The concept of health literacy, as a core indicator of an individual’s ability to manage their health, was first introduced by American scholar Simonds in 1974 at the International Health Conference in Bangkok (Simonds, 1974). Theoretically defined, health literacy encompasses an individual’s capacity to access, comprehend, and apply basic health information and services, enabling informed decisions conducive to maintaining and promoting their health (China Health Commission, 2016). This concept extends beyond cognitive skills to include the application of social skills, emphasizing the individual’s ability to actively access, understand, and utilize health information (Kutner et al., 2006). Globally, approximately 39% of individuals possess inadequate health literacy (Cajita et al., 2016). Within China, by the end of 2022, the population’s health literacy level was reported to be 27.78%, indicating that fewer than three out of ten individuals demonstrate adequate health literacy (Li Y. et al., 2024) Of particular concern is the college student population, where the Eighth National Research on Students’ Physical Fitness and Health highlights a concerning state of physical fitness, drawing significant attention from academic and societal spheres (Department of Physical Health and Arts Education, Ministry of Education, 2021a; Department of Physical Health and Arts Education, Ministry of Education, 2021b). Research demonstrates that health literacy levels are significantly correlated with individual health status. Low health literacy is often associated with an increase in negative psychological states, such as depression and anxiety, and a decline in quality of life (Berkman et al., 2011b; Smith et al., 2015). Conversely, high health literacy empowers individuals to maintain their health and promotes the adoption of positive health behaviors (Nutbeam and Nutbeam, 1986).”.

Life satisfaction, a significant indicator of an individual’s mental well-being, reflects their subjective evaluation of their life circumstances (Johnson, 1978). Building upon Michalos’ Multiple Discrepancies Theory (MDT), an individual’s life satisfaction is influenced by the perceived discrepancy between their actual and ideal state, with smaller discrepancies correlating with greater happiness (Michalos, 1985; Diener et al., 2009). From a social adaptation perspective, individuals with high life satisfaction tend to exhibit stronger social connections and more frequent pro-social behaviors (Diener and Seligman, 2002), fostering the establishment of robust interpersonal networks (Lyubomirsky et al., 2005); Furthermore, from an individual development standpoint, high life satisfaction is not only contributes to enhanced quality of life and improved physical and psychological well-being but also supports individuals in achieving greater success in academic pursuits, occupational endeavors, and interpersonal relationships (Antaramian, 2017; Schwarz and Strack, 1999). Positive life events and robust social support networks have also been identified as important factors in bolstering life satisfaction (Diener and Seligman, 2002; Cohen and Wills, 1985). Notably, research by Jaclyn et al. demonstrated that exercise, as a positive lifestyle modification, significantly enhances an individual’s life satisfaction (Maher et al., 2013). These findings underscore that life satisfaction is shaped by the interplay of subjective and objective factors and is closely intertwined with an individual’s behavioral patterns and social engagement. Consequently, exploring the influence mechanism of health literacy on life satisfaction holds significant theoretical and practical value.

This study employs the Health Belief Model (HBM) as its theoretical framework, a widely recognized psychosocial model utilized to explain and predict individual health behaviors. The HBM comprises five core constructs: perceived susceptibility, perceived severity, perceived benefits, perceived barriers, and cues to action. Perceived susceptibility represents an individual’s subjective assessment of their likelihood of contracting a disease; perceived severity reflects an individual’s awareness of the potential adverse consequences of a disease; perceived benefits encompass an individual’s appraisal of the positive outcomes associated with adopting health behaviors; perceived barriers pertain to an individual’s consideration of the obstacles that may be encountered in implementing health behaviors; and cues to action represent the various triggers that prompt an individual to adopt a health behavior. Within the framework of this study, health literacy serves as the independent variable, constituting the initiating factor within the causal chain. According to the HBM, an individual’s level of health literacy directly influences their perceptions and decision-making processes regarding health behaviors. Life satisfaction, designated as the dependent variable, represents the outcome variable of this study. Health literacy is hypothesized to indirectly influence life satisfaction through mediating variables. Specifically, physical exercise and sleep quality function as mediating variables, serving as intermediaries between health literacy and life satisfaction. These mediating variables are posited to be influenced by health literacy and, in turn, to exert an effect on life satisfaction, thereby establishing an indirect pathway through which health literacy affects the life satisfaction of college students.

**Figure 1:**
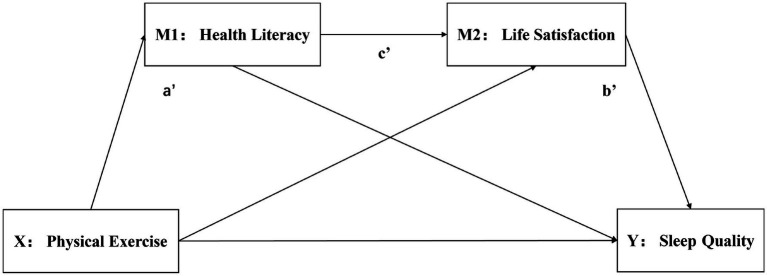
Diagram of the hypothetical mode.

Based on the theoretical framework, the following hypotheses are proposed, and the hypothetical model is presented in Figure 1:

*H1*: Physical exercise significantly positively predicts sleep quality.

*H2*: Health literacy significantly mediates the positive relationship between physical exercise and sleep quality. As shown in Figure 1, a’.

*H3*: Life satisfaction significantly mediates the positive relationship between physical exercise and sleep quality. As shown in Figure 1, b’.

H4: Health literacy and life satisfaction exhibit a significant serial mediating effect between physical exercise and sleep quality. As shown in Figure 1, c’.

## Methods

### Data sources

This study adheres to the American Psychological Association’s (APA) Ethical Principles of Psychologists and Code of Conduct.^1^ The survey was conducted using an anonymous completion format. The collection of demographic information for this study includes school, gender, and academic year. Before distributing the questionnaires, researchers read aloud the introductory section of the survey and informed participants of the relevant consent and completion requirements. Participants were provided with oral consent before receiving the questionnaires. After reviewing the introductory section of the survey once more, if participants wished to continue, they could proceed to complete it on a fully voluntary basis, and their data would be collected. Should participants be unwilling to fill out the survey, they had the option to discontinue without penalty, and their information would not be recorded.

This study utilized data from the ‘Chinese College Students Physical Activity and Health Longitudinal Survey (CPAHLS-CS) 2024’. The CPAHLS-CS is designed to generate a comprehensive, high-quality microdata set representing Chinese college students’ physical activity, physical health, and mental health behaviors. This dataset facilitates the analysis of relevant issues at the intersection of physical activity and health within this population, promoting interdisciplinary research on college student well-being. The CPAHLS-CS has been extensively employed to analyze the health status of Chinese college students (Zhu et al., 2024b; Zhu et al., 2024a; Zhu et al., 2024d; Qin et al., 2024; Mu et al., 2024a; Mu et al., 2024b; Li et al., 2024b; Li et al., 2024a; Li et al., 2022; Han et al., 2022c; Han et al., 2022a). The survey participants selected for this study comprised students enrolled in general higher education institutions within mainland China. The recruitment period for the study spanned from 08/10/2024 to 09/11/2024, with the subsequent questionnaire survey being conducted from 11/11/2024 to 24/11/2024. The list of eligible institutions was derived from the Ministry of Education’s ‘National List of General Higher Education Schools (as of 20 June 2024)’. Exclusion criteria were applied as follows: (1) Questionnaires failing to identify the full name of the respondent’s institution were removed. (2) Questionnaires exhibiting at least 21 consecutive identical entries, as indicated by their coded responses, were excluded. (3) Questionnaires with completion times falling within the lowest 0.5% and highest 0.5% of the distribution, relative to the average completion time of 6 min and 12 s (Zhu et al., 2024b), were discarded. The study sample was restricted to college students attending higher education institutions located in the central region of China (Anhui, Hunan, Hubei, and Jiangxi Provinces), resulting in a final sample size of 12,646 cases. The distribution of the sample is presented in Table 1.

**Table 1:** Sample distribution.

Variant	*n*	%
Gender		
Male	5,041	39.9
Female	7,605	60.1
Grade		
Freshman	8,697	68.8
Sophomore	3,536	28
Junior	337	2.7
Senior	76	0.6
(Grand) total	12,646	100

### Measuring tools

#### Sociological demographic information

Sociodemographic characteristics considered in this study encompassed gender, academic year, and related variables.

#### Physical exercise

Physical activity levels among college students were assessed using the Physical Activity Rating Scale-3 (PARS-3). (PARS-3) Developed by Japanese scholars Tokunaga and Hashimoto, and revised by the Chinese scholar Liang, it was used. The scale comprises 3 items, each rated on a 5-point scale, with total scores ranging from 0 to 100. Higher scores indicate greater physical activity. The scoring criteria are low activity (≤19 points), medium activity (20–42 points), and high activity (≥ 43 points). The calculation formula for physical exercise is shown as Equation 1 (Zhu et al., 2024c). Studies have shown that the scale has an internal consistency reliability of 0.856 and a test–retest reliability of 0.820.


(1) $$\begin{array}{l} \text{Physical Exercise}=\text{frequency of physical exercise}\\ \times \text{intensity of physical exercise}\;\\ \times (\text{duration of physical exercise}-1) \end{array}$$

The PARS-3 scores align with the classification of physical activity into light physical activity (LPA), moderate physical activity (MPA), and vigorous physical activity (VPA) within the Asian population. The resulting PARS-3 scores provide a measure of physical activity, reflecting, to some extent, the current status of university students’ physical activity engagement over a defined period. The PARS-3 demonstrates adequate test–retest reliability (0.820) and has been validated for use within the Chinese university student population across multiple studies.” (Mu et al., 2024a; Mu et al., 2024b; Li et al., 2022; Han et al., 2022c; Han et al., 2022a; Han et al., 2022b; Xu et al., 2023).

#### Sleep quality

Sleep quality among college students was assessed using the Pittsburgh Sleep Quality Index (PSQI). Developed in 1989 by Dr. Buysse and colleagues at the University of Pittsburgh, the PSQI is a validated instrument for evaluating sleep quality and monitoring treatment outcomes in individuals with sleep disorders and psychological conditions. It is also appropriate for investigating sleep quality in the general population and exploring the relationship between sleep quality and both mental and physical health. The scale has been adapted for the Chinese context by Liuto to enhance its applicability. The PSQI comprises 23 items, grouped into seven subscales, each rated on a scale from 0 to 3. The sum of the subscale scores yields a total PSQI score, ranging from 0 to 21, with higher scores indicating poorer sleep quality. Established cut-off scores for the PSQI are as follows: 0–4 indicates good sleep quality, 5–7 indicates fair sleep quality, and 8–21 indicates poor sleep quality. The PSQI demonstrates satisfactory reliability and validity, with a reported Cronbach’s alpha coefficient of 0.764 (Liu et al., 1996).

#### Health literacy

Health literacy among college students was assessed using the Health Literacy Scale (HLS-SF9). Health literacy encompasses not only traditional literacy skills but also a broader array of abilities, including numeracy, communication skills, and the capacity to interpret and critically evaluate health information. The HLS-SF9, a nine-item version, was derived from the original HLS-SF12 by Sun et al. (2022). This simplified health literacy scale demonstrates satisfactory reliability and validity, serving as an efficient tool for rapidly assessing health literacy within the Chinese population. Response options for each item are: “very difficult,” “difficult,” “simple,” and “very simple.” The HLS-SF9 exhibits neither ceiling nor floor effects, demonstrating a Cronbach’s alpha coefficient of 0.913 and a split-half reliability of 0.871. Empirical factor analysis of the HLS-SF9 yielded the following fit indices: χ^2^/df = 10.844, Goodness of Fit Index (GFI) = 0.985, Adjusted Goodness of Fit Index (AGFI) = 0.971, Normative Fit Index (NFI) = 0.986, Comparative Fit Index (CFI) = 0.987, and Root Mean Square Error of Approximation (RMSEA) = 0.051 (Sun et al., 2022).

#### Life satisfaction

The Satisfaction With Life Scale (SWLS) was utilized to measure the life satisfaction of university students. Developed in the 1980s by American scholars Diener and colleagues, the SWLS comprises five items rated on a 7-point scale from 0 to 6, with a total possible score of 30. The higher the sum of the five items, the stronger the sense of life satisfaction. A score of 20 serves as the critical value distinguishing dissatisfaction from satisfaction (Pavot and Diener, 2009). Chinese scholars Xiong Chengqing and colleagues conducted assessments of the reliability of the Satisfaction With Life Scale (SWLS) using SPSS 13.0, performing Cronbach’s alpha analysis for internal consistency and split-half reliability analysis. The results indicated an alpha coefficient of 0.78 and a split-half reliability of 0.70 for the life satisfaction scale. The reliability analysis demonstrated that the internal consistency reliability coefficient and split-half reliability of the life satisfaction scale are relatively high, suggesting that the scale has good reliability for measuring the life satisfaction of the general population. The aforementioned research findings indicate that the life satisfaction scale is an effective and reliable tool for assessing the life satisfaction of the general public.

### Statistical analyses

Data analysis for this study was conducted using SPSS 26.0 and Microsoft Excel. The analytical process comprised the following steps:

Initial data preprocessing was performed using Microsoft Excel to identify and address any missing or problematic data points. Questionnaires with incomplete or inconsistent responses were either corrected or removed.

To mitigate potential common method bias (CMB) arising from the use of self-report scales, Harman’s single-factor test was employed. Exploratory factor analysis (EFA) was conducted on all items about physical exercise, sleep quality, health literacy, and life satisfaction. The results yielded five principal components with eigenvalues exceeding 1, with the largest factor accounting for 25.9% of the variance. This value falls below the commonly accepted threshold of 40%, indicating that CMB is unlikely to significantly influence the findings of this study.

Differences in physical exercise and sleep quality across gender and academic year were analyzed using the chi-square test. Cramer’s *V* coefficient was employed to quantify the strength of association between these categorical variables, with values ranging from 0 to 1. A Cramer’s *V* coefficient greater than 0.3 suggests a moderate association, while a coefficient exceeding 0.5 indicates a strong association. Differences in health literacy and life satisfaction were analyzed using ANOVA, with effect sizes quantified using η^2^ (eta-squared), ranging from 0 to 1. Following Cohen’s guidelines, η^2^ values of 0.01, 0.06, and 0.14 were interpreted as small, medium, and large effects, respectively.

Pearson’s correlation analysis was utilized to examine the bivariate relationships among physical exercise, sleep quality, health literacy, and life satisfaction.

Mediation analyses were conducted using regression analyses, multiple regression analyses, and the PROCESS macro, employing the bootstrap method to assess the significance of indirect effects.

## Results

### Descriptive analysis

Table 2 presents the results of chi-square analyses, revealing no statistically significant differences in physical exercise and sleep quality among college students based on gender and academic year. Specifically, the chi-square test for physical exercise yielded a statistically significant result at the gender level (χ^2^ = 1671.295, *V* = 0.364) and the grade level (χ^2^ = 318.679, *V* = 0.112). Similarly, the chi-square test for sleep quality demonstrated statistically significant results at the gender level (χ^2^ = 32.518, V = 0.051) and the grade level (χ^2^ = 87.199, *V* = 0.048).

**Table 2:** Descriptive analysis results.

Variant		Statistical	Physical exercise			Sleep quality			
		Indicators	Low intensity	Medium intensity	High strength	Excellent	Not bad	Usual	Poorly
		*n*	9,015	2086	1,545	3,226	6,209	2,705	506
		%	71.3	16.5	12.2	25.5	49.1	21.4	4.0
Gender	Male	*n*	2,620	1,216	1,205	1,223	2,456	1,104	258
		%	52.0	24.1	23.9	24.3	48.7	21.9	5.1
	Female	n	6,395	870	340	2003	6,209	2,705	506
		%	84.1	11.4	4.5	26.3	49.3	21.1	3.3
			x*^2^* = 1671.295			x*^2^* = 32.518			
			*p* = 0.364			*p* = 0.051			
			V = 0.364			V = 0.051			
Grade	Freshman	*n*	6,195	1,501	1,001	2,386	4,270	1729	312
		%	71.2	17.3	11.5	27.4	49.1	19.9	3.6
	Sophomore	*n*	2,646	506	384	758	1747	858	173
		%	74.8	14.3	10.9	21.4	49.4	24.3	4.9
	Junior	*n*	146	63	128	64	160	96	17
		%	43.3	18.7	38.0	19.0	47.5	28.5	5.0
	Senior	n	28	16	32	18	32	22	4
		%	36.8	21.1	42.1	23.7	42.1	28.9	5.3
			x*^2^* = 318.679			x*^2^* = 87.199			
			*p* = 0.159			*p* = 0.083			
			*V* = 0.112			*V* = 0.048			

Table 3 presents the ANOVA results, indicating the following: At the gender level, the effect size for life satisfaction (η^2^ = 0) was considerably smaller than that for health literacy (η^2^ = 0.009), suggesting a greater influence of gender on health literacy compared to life satisfaction. In summary, both male (*M* = 22.704, SD = 5.920) and female (*M* = 22.622, SD = 5.295) students reported life satisfaction scores above the norm of 20, indicating a relatively high level of life satisfaction among Chinese university students. At the academic year level, the effect size was equivalent for both health literacy (η^2^ = 0.001) and life satisfaction (η^2^ = 0.001). Examining health literacy, the effect of academic year (η^2^ = 0.001) was smaller than the effect of gender (η^2^ = 0.009). Considering life satisfaction, both gender (η^2^ = 0) and academic year (η^2^ < 0.001) exhibited small effect sizes. With the increase in academic year, there is a corresponding rise in the level of life satisfaction among students, suggesting that Chinese university students generally exhibit a satisfactory level of life satisfaction.

**Table 3:** Descriptive analysis results.

Variant		Statistical indicators	Health literacy	Life satisfaction
		*M*	27.760	22.655
		sd	4.084	5.552
Gender	Male	*M*	28.225	22.704
		sd	4.519	5.920
	Female	*M*	27.452	22.622
		sd	3.737	5.295
	η^2^		0.009	<0.001
	F		109.493	0.656
	P		<0.001	0.418
Grade	Freshman	*M*	27.836	22.592
		sd	4.021	5.507
	Sophomore	*M*	27.566	22.702
		sd	4.209	5.616
	Junior	*M*	27.893	23.398
		sd	4.280	5.681
	Senior	*M*	27.500	24.316
		sd	4.219	6.710
	η^2^		0.001	0.001
	F		3.872	4.743
	P		0.009	0.003

### Correlation analysis

Table 4 presents the Pearson correlation coefficients, revealing the following relationships: A statistically significant, albeit weak, negative correlation was observed between physical exercise and sleep quality (*r* = −0.032). Sleep quality exhibited a significant negative correlation with health literacy and its sub-dimensions, with correlation coefficients ranging from −0.172 to −0.143. Physical exercise demonstrated a significant positive correlation with health literacy and its sub-dimensions, with correlation coefficients ranging from 0.149 to 0.218. Life satisfaction was significantly positively correlated with physical exercise (*r* = 0.137), significantly negatively correlated with sleep quality (*r* = −0.259), and significantly positively correlated with health literacy and its sub-dimensions, with correlation coefficients ranging from 0.302 to 0.363.

**Table 4:** Correlation analysis.

Variant	A	B	C	C_1	C_2	C_3	D
A_ Amount of physical activity	1						
B_ Sleep Quality	−0.032^**^	1					
C_ Health Literacy	0.198^**^	−0.172^**^	1				
C_1_Health care	0.149^**^	−0.143^**^	0.886^**^	1			
C_2_Disease prevention and control	0.167^**^	−0.160^**^	0.915^**^	0.720^**^	1		
C_3_Health Promotion	0.218^**^	−0.159^**^	0.886^**^	0.668^**^	0.725^**^	1	
D_ Life satisfaction	0.137^**^	−0.259^**^	0.363^**^	0.302^**^	0.340^**^	0.334^**^	1

**At the 0.01 level (two-tailed), the correlation is significant.

### Regression analysis

**Figure 2:**
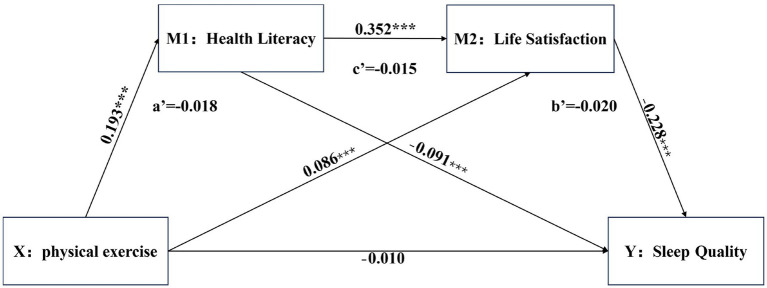
Path analysis diagram.

Mediation analyses were conducted using SPSS 27.0, employing Model 6 within the PROCESS macro and the bootstrap method. These analyses, controlling for gender and academic year, examined the chain-mediated effects of health literacy and life satisfaction on the relationship between physical exercise and sleep quality. The results, presented in Table 5, revealed that after controlling for gender and academic year, physical exercise negatively predicted sleep quality (*β* = −0.010). However, the relationship is not statistically significant (*p* > 0.001), thus, this study is characterized by complete mediation. Since a significant negative correlation was identified between physical exercise and sleep quality in the aforementioned correlation analysis, this study employed methods of multicollinearity diagnostics to assess the potential for high intercorrelations among the variables, which could otherwise lead to model instability. Upon examination, the Variance Inflation Factors (VIFs) for physical exercise, health literacy, life satisfaction, and sleep quality were all found to be less than 5, indicating that there is no issue of multicollinearity in this study (O’brien, 2007). The scoring mechanism of the Pittsburgh Sleep Quality Index (PSQI) is inversely oriented, meaning that lower scores suggest higher sleep quality for the participants; correspondingly, higher scores indicate poorer sleep quality. Consequently, the negative effect size implies that greater engagement in physical exercise is associated with poorer sleep quality, and conversely, lower levels of physical exercise are associated with better sleep quality. This finding indicates that increased physical exercise was associated with poorer sleep quality, as measured by the PSQI, while decreased physical exercise was associated with better sleep quality. Physical exercise positively predicted health literacy (*β* = 0.193, *p* < 0.001) and life satisfaction (*β* = 0.086, *p* < 0.001). Health literacy negatively predicted sleep quality (*β* = −0.091, *p* < 0.001) and positively predicted life satisfaction (*β* = 0.352, *p* < 0.001). Finally, life satisfaction negatively predicted sleep quality (*β* = −0.228, *p* < 0.001), indicating that higher life satisfaction was associated with better sleep quality, while lower life satisfaction was associated with poorer sleep quality, as reflected in the PSQI. The mediation model is illustrated in Figure 2.

**Table 5:** Results of regression analyses excluding confounders.

Regression equation		Overall fit index			Significance of regression coefficients			VIF
Outcome variable	Predictor variable	*R*	*R* ^2^	*F*	*β*	SE	*t*	
Sleep quality		0.29	0.084	231.379***				
	Physical exercise				−0.010	0.009	−1.086	1.209
	health literacy				−0.091	0.009	−9.795***	1.187
	Life satisfaction				−0.228	0.009	−24.790***	1.162
	Gender				−0.115	0.019	−6.158***	1.160
	Grade				0.150	0.015	9.835***	1.011
Health literacy		0.203	0.041	180.261***				
	Physical exercise				0.193	0.090	20.452***	
	Gender				−0.045	0.008	−2.328***	
	Grade				−0.065	0.016	−4.195***	
Life satisfaction		0.374	0.14	512.898***				
	physical exercise				0.086	0.009	9.466***	
	health literacy				0.352	0.008	41.747	
	Gender				0.114	0.018	6.317***	
	Grade				0.048	0.015	3.237***	

***P < 0.001. If there is no asterisk (***), it indicates that *p* > 0.001.

### Tests for mediating effects

As presented in Table 6, the 95% confidence interval (CI) for the indirect effect of physical exercise on sleep quality through health literacy was [−0.022, −0.013], excluding 0. This finding indicates that health literacy significantly mediated the relationship between physical exercise and sleep quality. The 95% CI for the indirect effect of physical exercise on sleep quality through life satisfaction was [−0.024, −0.015], also excluding 0, supporting the mediating role of life satisfaction in this relationship. Furthermore, the 95% CI for the chain-mediated effect of health literacy and life satisfaction on the relationship between physical exercise and sleep quality was [−0.018, −0.013], confirming the presence of a significant chain-mediated effect.

**Table 6:** Results of mediation effects analysis.

Variant	Efficiency value	*BootSE*	*LLCI*	*ULCI*
Aggregate effect	−0.630	0.010	−0.080	−0.044
Direct effect	−0.010	0.009	−0.029	0.008
Indirect effect	−0.053	0.004	−0.059	−0.046
Amount of physical activity→ Health literacy→ Sleep Quality	−0.018	0.002	−0.022	−0.013
Amount of physical activity→ Life satisfaction→ Sleep Quality	−0.020	0.003	−0.024	−0.015
Amount of physical activity→ health literacy→ life satisfaction→ sleep quality	−0.015	0.001	−0.018	−0.013

## Discussion

As presented in Table 5, the 95% confidence interval (CI) for the indirect effect of physical exercise on sleep quality through health literacy was [−0.022, −0.013], excluding 0. This finding indicates that health literacy significantly mediated the relationship between physical exercise and sleep quality. The 95% CI for the indirect effect of physical exercise on sleep quality through life satisfaction was [−0.024, −0.015], also excluding 0, supporting the mediating role of life satisfaction in this relationship. Furthermore, the 95% CI for the chain-mediated effect of health literacy and life satisfaction on the relationship between physical exercise and sleep quality was [−0.018, −0.013], confirming the presence of a significant chain-mediated effect.

There is no significant positive predictive relationship between physical exercise and sleep quality. This study did not find a significant positive predictive relationship between physical exercise and sleep quality among college students, failing to support hypothesis H1. This study demonstrates a complete mediation effect (Baron and Kenny, 1999), where the impact of physical exercise on sleep quality is mediated through the pathways of health literacy and life satisfaction. The relationship between physical exercise and sleep quality remains inconclusive within the academic literature. This finding contradicts our initial hypothesis and warrants further investigation into the underlying mechanisms. To validate the robustness of our regression model, we performed multicollinearity diagnostics. Multicollinearity, which occurs when predictor variables exhibit high intercorrelations, can compromise the precision of coefficient estimates and undermine model stability (O’brien, 2007). We assessed this using VIFs, where values exceeding 5–10 typically indicate problematic multicollinearity (Nordhausen Review, 2014). In our analysis, all VIFs values remained below the conservative threshold of, confirming the absence of substantial multicollinearity and supporting the reliability of our regression estimates. Notably, these results suggest that for college students, physical exercise may not directly influence sleep quality but operates through indirect pathways. Specifically, our mediation analysis indicates that physical activity might enhance sleep quality by improving health literacy and life satisfaction, serving as intermediary mechanisms in this relationship. This mediated pattern aligns with contemporary theoretical frameworks positing that health behaviors often affect outcomes through psychosocial pathways rather than direct effects (Baron and Kenny, 1999). While some scholars suggest that physical activity may positively impact sleep quality, they also acknowledge the absence of a significant mediating effect in some cases. This may be attributed to the multifaceted nature of sleep quality, influenced by genetic factors, lifestyle habits, and psychological state, potentially masking the direct effect of physical activity (Uchida et al., 2012; Driver and Taylor, 2000). Conversely, other studies have demonstrated a significant positive correlation between regular, high-intensity, or moderate-intensity physical activity and sleep quality (Brand et al., 2010; Chennaoui et al., 2015; Gerber et al., 2014), Specifically, physical activity may improve sleep quality through mechanisms such as regulating biological rhythms and alleviating stress and anxiety (Chennaoui et al., 2015). Regular physical activity has been associated with shorter sleep onset, fewer night awakenings, and higher sleep efficiency (Brand et al., 2010), leading to advantages in sleep quality, mental health, and stress management (Gerber et al., 2014). While the benefits of physical activity on physical and mental health are well-established (Matheson, 2020; MCSP PDJJP, 2002; Schuch et al., 2018; Nieman and Wentz, 2019; Benhaberou-Brun, 2012). The insignificant effect of physical activity on sleep quality in this study may be due to variations in physical activity types and measurement methods. The lack of a significant mediating effect may indicate that not all forms of physical activity are equally associated with sleep quality. Future research should further investigate the impact of different types, intensities, and frequencies of physical activity on sleep quality to clarify this complex relationship.

### Negative mediating effects of health literacy

This study revealed a significant negative mediating role of health literacy in the relationship between physical exercise and sleep quality, the scoring mechanism of the Pittsburgh Sleep Quality Index (PSQI) is inversely oriented, meaning that lower scores indicate better sleep quality for participants; correspondingly, higher scores suggest poorer sleep quality. Consequently, the effect size is negative, indicating that greater levels of physical exercise are associated with worse sleep quality, and vice versa, thus supporting hypothesis H2. The findings suggest that the negative mediating effect of health literacy further reinforces the positive impact of physical exercise on sleep quality. This result aligns with previous research demonstrating significant mediating effects between health literacy levels and both physical exercise and sleep quality. Specifically, individuals with lower health literacy often exhibit less regular exercise habits and a higher propensity for sleep disturbances, whereas those with higher health literacy tend to maintain regular physical activity and report better sleep quality (Berkman et al., 2011a; Ishikawa et al., 2008). Furthermore, individuals with high health literacy are more proactive in adopting healthy lifestyles, including maintaining a regular schedule and engaging in physical activity (Sorensen et al., 2012). It is important to acknowledge the bidirectional relationship between physical exercise and health literacy. Higher levels of physical activity are often associated with increased health literacy, as individuals are more likely to actively seek, understand, and apply health-related information. Conversely, higher health literacy can encourage participation in physical activity, creating a mutually reinforcing cycle (Ishikawa and Yano, 2008). The combined effect of physical activity and health literacy on sleep quality highlights the potential benefits of comprehensive interventions that integrate both approaches. Such integrated interventions may optimize sleep quality in a multidimensional manner by improving physical fitness and health literacy (Reid et al., 2010). These results emphasize the critical role of health literacy as a mediator, which, in turn, enhances and sustains the positive influence of physical exercise, fostering the development of healthy lifestyles. Based on these findings, strengthening health literacy interventions is crucial for enhancing the quality of life and promoting the physical and mental well-being of college students. By improving health literacy, we can encourage participation in regular physical exercise and effectively improve sleep quality, thus contributing to overall well-being and safeguarding physical and mental health.

### Negative mediating effects of life satisfaction

This study revealed a significant negative mediating effect of life satisfaction on the relationship between physical exercise and sleep quality. The scoring mechanism of the Pittsburgh Sleep Quality Index (PSQI) is inversely proportional, such that lower scores signify higher sleep quality for participants; conversely, higher scores indicate poorer sleep quality. Therefore, the effect size is negative, implying that increased physical exercise correlates with decreased sleep quality, and the converse is also true. Thus supporting hypothesis H3. Furthermore, significant positive correlations were observed between life satisfaction and health literacy, as well as its sub-dimensions, and a significant negative correlation with sleep quality. The results suggest that, within the context of physical activity, the enhancement of life satisfaction among college students significantly and positively contributes to their sleep quality. This finding aligns with prior research demonstrating that physical activity directly improves sleep quality and indirectly optimizes sleep quality by increasing life satisfaction. Notably, this mediating effect was particularly pronounced among college students with sustained participation in physical activity (Biddle and Asare, 2011). Hamer et al. (2009) found that individuals engaged in regular physical activity typically reported higher levels of life satisfaction, and this elevated satisfaction was strongly associated with the psychological benefits of physical activity, such as stress relief and mood regulation (Hamer et al., 2009). Consequently, increased life satisfaction can effectively mitigate the negative effects of anxiety and depression on sleep, leading to better sleep quality among individuals with higher life satisfaction (Steptoe et al., 2008). Physical exercise is widely recognized as an effective means of stress reduction and emotion regulation, especially for the college student population, which is particularly susceptible to sleep disorders due to academic pressures and social adjustment challenges. The study by Kalak et al. (2012) further confirmed that college students participating in physical exercise exhibited significantly higher life satisfaction and superior sleep quality compared to non-exercising students. This supports the notion that physical exercise offers an effective pathway for college students to improve sleep quality by enhancing life satisfaction (Kalak et al., 2012). Moreover, the cumulative effect of long-term exercise on both life satisfaction and sleep quality was particularly significant, further supporting the mediating role of life satisfaction (Reid et al., 2010). The positive impacts of life satisfaction extend beyond improvements in an individual’s well-being and physical fitness; it also serves as a pathway to enhance overall quality of life. By increasing life satisfaction, individuals can achieve comprehensive improvements in physical and mental health, emotional regulation, and social adaptation, fostering a virtuous cycle that further enhances overall quality of life.

The serial mediating role of health literacy and life satisfaction. This study demonstrated that health literacy and life satisfaction significantly mediated the relationship between physical exercise and sleep quality. The scoring mechanism of the Pittsburgh Sleep Quality Index (PSQI) is inversely proportional, such that lower scores signify higher sleep quality for participants; conversely, higher scores indicate poorer sleep quality. Therefore, the effect size is negative, implying that increased physical exercise correlates with decreased sleep quality, and the converse is also true. Supporting research hypothesis H4. The correlation analyses revealed significant positive correlations among health literacy, its sub-dimensions, life satisfaction, and physical exercise. Conversely, significant negative correlations were observed between health literacy, its sub-dimensions, life satisfaction, and sleep quality. These findings suggest that physical exercise positively influences health literacy, which, in turn, enhances life satisfaction, ultimately leading to improved sleep quality among college students.

This study’s findings can be interpreted through the lens of Self-Determination Theory (SDT), proposed by Deci and Ryan, which posits that human behavior is driven by three fundamental psychological needs: autonomy, competence, and relatedness. SDT suggests that fulfillment of these needs enhances intrinsic motivation and well-being, fostering positive behaviors and improved health outcomes (Deci and Ryan, 2008). Physical exercise, a key variable in this study, is closely linked to these basic psychological needs. Research indicates that participation in physical activity enhances individuals’ autonomy (through the selection of preferred activities), competence (by developing physical skills and fitness), and relatedness (through social interaction in team sports or exercise groups). Teixeira et al. (2012) demonstrated that physical activity that fulfills these basic psychological needs significantly improves individuals’ participation and adherence to exercise. Furthermore, Deci and Ryan (2008) noted that individuals whose basic psychological needs are met are more likely to develop higher levels of health literacy, which, in turn, promotes health behaviors such as improved sleep quality (Deci and Ryan, 2008). Autonomy fosters proactive health learning through autonomous exercise choices; competence is reflected in the sense of achievement gained through exercise, boosting confidence in health management; and social support from coaches or peers facilitates access to health information. Life satisfaction, a key indicator of well-being within SDT, mediates the relationship between fulfilling basic psychological needs and positive health outcomes (Huta and Ryan, 2010). This study’s findings support the notion that physical activity significantly enhances individuals’ life satisfaction by satisfying these basic psychological needs. The subsequent improvement in sleep quality, a crucial outcome variable, is indirectly achieved through physical activity that meets basic psychological needs. Specifically, individuals with high health literacy and life satisfaction tend to adopt healthier behaviors and exhibit lower levels of stress and anxiety, thus promoting better sleep quality, a result consistent with Kredlow et al. (2015, p. 80). Synthesizing these observations through the framework of SDT, physical activity enhances health literacy and life satisfaction by fulfilling basic psychological needs, with these factors acting as mediating variables to further promote better sleep quality. Future studies could explore interventions aimed at maximizing the mediating roles of health literacy and life satisfaction (e.g., by enhancing the satisfaction of basic psychological needs) to optimize sleep quality and overall health among university students.

The HBM and SDT jointly underpin the overall framework of the article. The HBM elucidates how health literacy influences individuals’ perceptions and behavioral decisions regarding physical exercise, while the SDT explains from a motivational perspective how physical exercise can fulfill fundamental psychological needs, thereby enhancing life satisfaction and sleep quality. These theoretical models not only substantiate the indirect impact of physical exercise on sleep quality but also provide a theoretical basis for future interventions aimed at improving sleep quality through enhancing health literacy and life satisfaction. The complete mediation model of this study emphasizes the crucial role of health literacy and life satisfaction in improving sleep quality among college students.

This study has several limitations: (1) The assessment of physical exercise and sleep quality relied solely on self-reported recall scales, without objective measurement. (2) The analysis did not examine the variable of physical exercise categorically, potentially limiting the comprehensiveness of the findings.

## Conclusion

In conclusion, this study empirically demonstrates that physical exercise indirectly influences sleep quality via a chain-mediated pathway involving health literacy and life satisfaction. This finding offers valuable data to support major universities in their efforts to improve students’ sleep quality and enhance their overall well-being.

## Data Availability

The raw data supporting the conclusions of this article will be made available by the authors, without undue reservation.

## Footnotes

^1^ http://www.apa.org/ethics/code/index.aspx
